# Preterm Birth, Kidney Function and Cardiovascular Disease in Children and Adolescents

**DOI:** 10.3390/children9081130

**Published:** 2022-07-28

**Authors:** Athanasia Chainoglou, Katerina Chrysaidou, Vasilios Kotsis, Stella Stabouli

**Affiliations:** 1 1st Department of Pediatrics, School of Medicine, Faculty of Health Sciences, Aristotle University Thessaloniki, Hippokratio Hospital, 54642 Thessaloniki, Greece; nchainog@gmail.com (A.C.); cathchris84@hotmail.com (K.C.); 2 Hypertension-24h ABPM ESH Center of Excellence, 3rd Department of Medicine, Aristotle University of Thessaloniki, Papageorgiou Hospital, 56429 Thessaloniki, Greece; vkotsis@auth.gr

**Keywords:** preterm, prematurity, kidney function, hypertension, cardiovascular disease, children

## Abstract

Over recent decades, there has been a global increase in preterm birth rate, which constitutes about 11% of total births worldwide. The present review aims to summarize the current knowledge on the long-term consequences of prematurity on renal and cardiovascular development and function. Recent literature supports that prematurity, intrauterine growth restriction or low birth weight (LBW) may have an adverse impact on the development of multiple organ systems, predisposing to chronic diseases in childhood and adulthood, such as arterial hypertension and chronic kidney disease. According to human autopsy and epidemiological studies, children born preterm have a lower nephron number, decreased kidney size and, in some cases, affected renal function. The origin of hypertension in children and adults born preterm seems to be multifactorial as a result of alterations in renal, cardiac and vascular development and function. The majority of the studies report increased systolic and diastolic blood pressure (BP) in individuals born preterm compared to full term. The early prevention and detection of chronic non-communicable diseases, which start from childhood and track until adulthood in children with a history of prematurity or LBW, are important.

## 1. Introduction

Over recent decades, there has been a global increase in preterm birth rate, which constitutes about 11% of the total number of births worldwide [1]. There is significant variation in the incidence of preterm birth worldwide. The rates of preterm birth in 2010 ranged from 5% in several Northern European countries to 18% in Malawi. More than 60% of all preterm births worldwide occur in low-income countries, such as sub-Saharan Africa and South Asia, as a result of the high live birth rates and high fertility in those regions. Notably, the United States of America (USA) is the only high-income country that ranks in the top ten countries with the greatest overall number of preterm births per year [2]. Preterm birth is defined as a live birth that occurs before 37 completed weeks of gestation [3]. Low birth weight (LBW) neonates are defined as neonates with a birth weight below 2500 g. Nearly 15% of total births worldwide are born with a low birth weight. The prevalence of low birth weight is estimated at about 2.5–8.5% across European countries and at about 8% in the USA [4]. Prematurity is the second leading cause of death in children under the age of 5 years and a significant cause of long-term health problems and impairments. There are significant differences in survival rates for preterm neonates born in high and low-resource settings due to disparities in availability and quality of provided healthcare services. In high-resource settings, approximately 50% of neonates born at 24 weeks survive, whereas in low-resource settings, over 90% of neonates born at less than 28 weeks will not survive the first week of life, and only half of those born at 32 weeks will survive [1,2]. Prematurity interrupts normal organogenesis and the development of tissues and organs, increasing the risk of developing chronic diseases during childhood and adulthood, such as chronic kidney disease (CKD), hypertension (HTN) or cardiovascular disease (CVD) [3,5]. In large population-based cohort studies, preterm birth was associated with a 53% increased relative risk of ischemic heart disease and a twofold increase in the risk of developing cerebrovascular disease and chronic kidney disease in adulthood [6,7].

First, Barker observed an inverse association between birth weight and the incidence of CVD, such as ischemic heart disease, stroke and hypertension during adulthood. He advanced the question of “developmental programming”, that is, whether the intrauterine environment and the stress that is experienced during fetal and early postnatal life increase the risk of later-life chronic diseases, such as hypertension and chronic kidney disease [8]. Since then, numerous studies have investigated the impact of prematurity, intrauterine growth restriction (IUGR) and LBW on kidney function and blood pressure (BP) during childhood and adulthood. The present review aims at reviewing the current knowledge on the long-term consequences of prematurity on renal and cardiovascular development and function.

## 2. Prematurity and Kidneys

Nephrons are the functional units of kidneys whose formation begins at the ninth gestational week. The majority of nephrons are developed during the third trimester of pregnancy and complete at 34–36 weeks of gestation [9]. The total number of nephrons varies from 200,000 to 2,000,000 per kidney, per person [10,11]. This wide variance may be due to either genetic or environmental factors that affect nephrogenesis prenatally, or harmful factors that may lead postnatally to loss of nephrons in later life. Maternal diseases, either developed in the pre-pregnancy stage (CKD and HTN) or during pregnancy (preeclampsia/eclampsia, gestational diabetes, chorioamnionitis), maternal habits (alcohol consumption, cigarette smoking, nutrition) or administrated medications to the pregnant mother (antenatal steroids) are factors that can affect prenatally the development of the kidney. They may either lead directly to low nephron endowment or indirectly by predisposing to preterm birth or IUGR and LBW [12,13]. A linear relationship has been observed between nephron number and birth weight, predicting an increase of 257,426 glomeruli per kilogram increase in birth weight [11]. Due to incomplete nephrogenesis, preterm infants are vulnerable to factors that affect kidney development in extrauterine life and can lead to renal injury. Such factors are administrated nephrotoxic medications (aminoglycoside antibiotics, non-steroidal anti-inflammatory drugs) and acute kidney injury during the neonatal period [12,13]. Moreover, it is known that the nephron number declines with age in a normal population without renal disease at a rate of approximately 4500 glomeruli per kidney, per year [14,15]. Finally, the nephron number tends to be lower in females [16].

According to human post-mortem autopsy studies, glomerulogenesis is markedly decreased in preterm infants, and the extra-uterine nephrogenesis continues to a maximum of 40 days after birth by the development of abnormal glomeruli without achieving a full complement of nephrons [17,18,19]. As a result, preterm infants are characterized by low nephron endowment, which leads to compensatory glomerular hyperfiltration by the remaining nephrons to minimize the overall reduction and loss of function, according to Brenner’s theory. Eventually, hyperfiltration results in the development of proteinuria, secondary focal segmental glomerulosclerosis and progressive decline of glomerular filtration rate (GFR) [20].

### 2.1. Prematurity and Renal Size during Childhood and Adulthood

Since there is no non-invasive method for correlating nephron number with kidney mass, many authors have used kidney volume as a surrogate marker (Table 1) [21,22,23,24,25,26,27,28,29,30,31,32,33,34,35,36]. Several studies have observed that infants and children born preterm had significantly smaller kidney volume or size, as estimated by renal ultrasound, compared with age-matched full-term individuals [22,26,27,29,30,32,33,35,37,38,39]. Similarly, studies that examined the effect of preterm birth on kidney size in adulthood reported significantly decreased kidney length and volume [23,28,31].

### 2.2. Prematurity and Renal Function during Childhood and Adulthood

However, findings from the studies that have examined long-term renal function after preterm birth are less conclusive. The parameters that have been examined in published literature are microalbuminuria as an early sign of hyperfiltration, albuminuria, levels of blood urea nitrogen, serum creatinine, serum cystatin C, estimated GFR (eGFR), impairments in electrolyte excretions and newer biomarkers of renal dysfunction, such as serum or urine neutrophil gelatinase-associated lipocalin (NGAL), symmetric dimethylarginine (SDMA) and serum b2 microglobulin (Table 1) [21,23,24,25,27,29,30,31,32,33,34,35,36,40,41,42,43,44,45]. There are a few studies on school-aged children and adolescents that did not observe any difference in eGFR between ex-preterm and term children [24,25,42]. On the other hand, the majority of the studies reported a significant decrease in eGFR or increase in albumin excretion in preterm children [21,36,41,46]. Moreover, other biomarkers, such as serum cystatin C or NGAL, have been reported to be higher in ex-preterm or extremely low birth weight children compared to term-born children [29,30,41,44]. Starzec et al. observed in a prospective 4-year follow-up study significantly higher levels of cystatin C, but within normal range, and lower eGFR in extremely low birth weight (ELBW) preterm children at 7 years of age. In the same study, cystatin C further increased above the normal range, and eGFR further decreased at the second follow-up at 11 years of age. These findings indicate that there is a tendency toward worsening renal function in ELBW children with age and suggest that these children may need further follow-up [30]. According to the results of a recent meta-analysis, preterm children and adults presented significantly decreased kidney length and volume, GFR and increased microalbuminuria, whereas serum levels of blood urea nitrogen, creatinine and cystatin C did not present any significant difference between preterm and full-term individuals. This finding indicates that traditional biomarkers may not reflect mild renal impairment as long as the renal function is maintained by the remained nephrons [47]. In another meta-analysis, children and adults with LBW had approximately 70% greater risk of developing CKD in later life, which was defined as albuminuria, low eGFR or end-stage renal disease (ESRD) [48]. Finally, national population-based studies in adults showed that there is an increased risk for the development of ESRD in adults with a perinatal history of prematurity, LBW or IUGR [49,50,51]. Crump et al. have reported that preterm birth was associated with a twofold risk for CKD from birth to mid-adulthood in both sexes [51]. Most of the studies tend to conclude that there is an impairment in the development and the function of the kidneys of preterm or IUGR infants. Thus, prematurity and IUGR may be recognized as risk factors for the manifestation of kidney dysfunction through childhood and adulthood, and the ex-preterms should be followed up for the rest of their life.

## 3. Prematurity and Cardiovascular Disease

The origin of HTN in children and adults born preterm may possibly be multifactorial as a result of alterations in renal, cardiac and vascular development and function of the neuroendocrine system. As mentioned above, according to Brenner’s theory, preterm birth leads to low nephron endowment and diminished filtration surface area, which results in restraint sodium excretion and elevated BP [20]. Moreover, various studies have reported alterations in different pathways of the renin-angiotensin system, which may contribute to the increase in BP [46,52,53]. Differences in the cardiac structure and function, such as increased left ventricular mass and significant reductions in systolic and diastolic functional parameters, have been reported in individuals born preterm. Moreover, the left ventricular mass was inversely related to gestational age, independently of BP levels [54]. Studies propose that the disruption of normal vasculogenesis in preterm children leads to structural and functional alterations of the vessels and impairment of endothelium-dependent vasodilatation, which lead to endothelial dysfunction and arterial HTN [55]. Furthermore, the oxidative stress and chronic inflammation of the tissues due to preterm birth and epigenetic mechanisms may be responsible for the increased risk of developing cardiovascular disease in later life [56]. The studies’ findings about the association of pulse wave velocity (PWV), as a marker of arterial stiffness, and preterm birth, are rather conflicting (Table 2) [57,58,59,60,61,62,63,64,65,66,67,68,69]. In a survey by Boardman et al., including a population sample of 102 ex-preterm adults and 102 controls, arterial stiffness was assessed by three different methods (carotid-femoral PWV, branchial-femoral PWV and cardiovascular magnetic resonance PWV) and did differ in association with a preterm perinatal history [64]. Notably, the number of studies in children is limited, and those that noticed that prematurity affects PWV found that among preterm children, mainly those with IUGR had significantly higher arterial stiffness [58,62]. We recently published a cross-sectional study assessing BP status by ambulatory BP monitoring and arterial stiffness by PWV in preterm children and adolescents showing higher nocturnal BP and increased arterial stiffness in preterm individuals compared to matched controls during childhood and adolescence. Preterm children who were overweight presented the highest values of night systolic BP and PWV levels [69]. Another possible mechanism, which might be responsible for HTN in preterm children, is the overactivation of the sympathetic system, which has been reported in a few studies on children born preterm or with low birth weight. Preterm and IUGR children displayed either increased catecholamine excretion or increased resting and stress-induced heart rate, which are consistent with sympathetic overactivation [70,71,72].

### 3.1. The Association of Prematurity with Hypertension during Childhood and Adulthood

Over recent decades, the impact of prematurity or intrauterine fetal growth in the development of hypertension during childhood and adulthood has been a significant topic of interest in several studies. A recent meta-analysis showed that being born preterm was associated with 3.26 mmHg higher mean systolic BP and 1.32 mmHg higher mean diastolic BP compared to being born at term. Subgroup analyses demonstrated that higher systolic BP was identified from early adolescence until adulthood among those born preterm and that the difference in systolic BP was greater in the extreme and very preterm born individuals compared to term [73]. In other meta-analyses, where BP was measured by both single-time office and ambulatory BP monitoring, preterm born adults compared to full term presented significantly higher systolic BP (mean difference: 4.2 mmHg), diastolic BP (mean difference: 2.6 mmHg) and 24 h ambulatory systolic BP (difference: 3.1 mmHg) [47,74,75]. Moreover, another meta-analysis reported additional differences in both sleeping and awake systolic BP and diastolic BP levels between the groups of preterm and term and significantly higher values of other components of metabolic syndromes, such as fasting glucose, insulin, Homeostasis Model Assessment-Estimated Insulin Resistance Index and total cholesterol levels in the group of preterm [75].

Several studies that examined kidney size, renal function and BP in children and adults have shown that preterm had smaller kidney size, impaired renal function and elevated BP compared to term [31,32,47]. Notably, Paquette et al. reported an inverse association of BP with kidney size in preterm individuals [31].

### 3.2. The Importance of Early Detection of Hypertension in Individuals Born Preterm

Even if the mean differences in BP seem insignificant between preterm and full-term children, the clinical implications are neglectable. Even a 2 mmHg reduction in BP is associated with 10% lower mortality from stroke and 7% lower mortality from ischemic heart disease in middle-aged adults [76]. Moreover, it is well known that systolic BP and diastolic BP may track from childhood to adulthood [77]. Overweight and weight changes are likely to affect BP tracking [78]. Specifically, in preterms and children with LBW, the accelerated weight gain in early childhood (1–5 years) has been associated with higher systolic BP in adulthood [79,80,81]. Hence, there are many risk factors that can act additionally to prematurity throughout life in the development of HTN and CVD, which could be prevented.

In conclusion, preterm birth is truly a global problem, with a high burden on both the poorest and the richest countries. According to reliable trend data on 65 countries, there has been an increase in preterm birth rates over the past 20 years in the majority of these countries. This increase may be due to the improved gestational assessment and the improved registration of most preterm neonates among countries. It may, however, represent a true increase because of health issues, such as an increase in maternal age, underlying maternal health problems, especially with increasing age of pregnancy, wider use of infertility treatments and increased rates of multiple pregnancies. The understanding of the real causes of the increase in preterm birth rate will advance the development of prevention solutions [82]. Preterm birth can result in a range of long-term complications in survivors, and the evidence, especially on the adverse impact of preterm birth and LBW on later kidney and CVD outcomes, is increasing. Most of the studies support that children born preterm have lower nephron number, decreased kidney size and, in some cases, affected renal function during childhood and adulthood. Moreover, children born preterm present increased systolic and diastolic blood pressure compared to those born full term. Children and adults with a perinatal history of IUGR, prematurity or LBW may require lifelong follow-ups of BP and renal function. Finally, the families of preterm or LBW children should be aware of the importance of a healthy lifestyle, as well as the importance of avoiding rapid weight gain during infancy and early childhood, as obesity may exacerbate the risk of future morbidity from kidney and cardiovascular diseases, as is also highlighted in the consensus statement of “Low Birth Weight and Nephron Number Working Group” [5].

## Figures and Tables

**Table 1:** Studies examining the association of prematurity with renal size, renal function and hypertension during childhood and adulthood.

Author, Year	Gestational Age at Birth for Cases	Age at Assessment	Study Groups (=Sample Size)	Outcome of Renal Size (RL or RV)	Outcome of Renal Function				Outcome of Blood Pressure Measurement
					sCreatinine	eGFR	uProtein (Albumin/Microalbumin)	Other Parameters of Renal Function	
Rodriquez Soriano et al., 2005 [21]	23–25 weeks	6.1–12.4 years	ELBW (=40) Control (=43)	No difference	Higher in ELBW	Lower in ELBW	No difference	uPhosphorus, uCalcium excretion: higher in ELBW	Office BP: no difference
Schmidt, 2005 [22]	<37 weeks	0 (first 5 days), 3, 18 months	Preterm (=178) Term (=717)	Smaller in preterm					
Keijzer-Veen et al., 2007 [23]	<32 weeks	20 years	Very Preterm (=51) (SGA:23, AGA:29), Term (=30)	Smaller in preterm SGA		Lower in preterm SGA. No difference after adjustment for BSA			Office BP: SBP: higher in preterm, DBP: no difference
Iacobelli et al., 2007 [24]	29.7 ± 2.5 weeks	6.3–8.2 years	VLBW-Preterm (=48) Term (=46)	No difference	No difference	No difference			Office BP: no difference
Rakow et al., 2008 [25]	<32 weeks	9–12 years	Preterm (=39), Term SGA (=29), Term AGA (=37)	Smaller in preterm	No difference	No difference	No difference	sCystatin: no difference	Office BP: no difference
Drougia et al., 2009 [26]	28–34, 34–46 weeks	0, 3, 6, 12, 24 months	28–34 weeks (=154) (SGA = 100, AGA = 54) 34–36 weeks (=141) (SGA = 80, AGA = 61) >36 weeks (=171) (SGA = 81, AGA = 90)	Smaller in preterm (28–34 weeks) at all ages and in near-term (34–36 weeks) after first 6 months.					
Baccheta et al., 2009 [27]	<30 weeks	7.6 years	Preterm (=50) (IUGR = 23, EUGR = 16) Control (=11)	Smaller in preterm		Lower in growth retarded		b-2 microglobulinuria:17% of preterm	BP: no difference
Keijzer-Veen et al., 2010 [28]	<32 weeks	20 years	Very Preterm (=51) (SGA:22, AGA:29), Term (=30)	Smaller in preterm					
Kwinta et al., 2011 [29]	26–29 weeks	6.7 years	ELBW (=78) Term (=38)	Smaller in ELBW			No difference	sCystatin:higher in ELBW	24 ABPM: no difference
Starzec et al., 2016 [30]	25–28 weeks	7, 11 years	ELBW (=64), Term (=36)	Smaller in ELBW at all ages	No difference			sCystatin: higher in ELBW at all ages, sBUN: higher in ELBW at 11 years	
Vashista et al., 2017 [40]	<30 weeks	30 (16–48) months	Preterm (=55) Term (51)				No difference	uphosphate/ Creatinine: higher in preterm, calcium excretion:no difference	Office BP: SBP, DBP: higher in preterm
Paquette et al., 2018 [31]	<29 weeks	23.2 years	Preterm (=92), Term (=92)	Smaller in preterm	No difference	No difference	Higher in preterm		24 ABPM: SBP, DBP, awake SBP: higher in preterm.
Gilarska et al., 2019 [32]	23–33 weeks	11 years	Preterm-ELBW (=157) Term (=123)	Smaller in preterm		Lower in ELBW			Office BP: SBP, DBP: higher in ELBW
Rakow et al., 2019 [33]	<28 weeks	7.7 years	Preterm with nephrocalcinosis (=20), preterm without nephrocalcinosis (=20), Term (=19)	Smaller in preterm		Lower in preterm, but normal			24 ABPM: no difference
Li et al., 2019 [34]	30–32 weeks	Birth, 37 weeks, 6 months	Preterm (=62) Term (=27)	Smaller in preterm at 37 weeks No difference at 6 months				sCystatin: higher in preterm at 37 weeks, no difference at 6 months	
Vollsaeter et al., 2018 [41]	24–31 weeks	11 years	Preterm-ELBW (=57) (SGA = 20, AGA = 37) Term-AGA (=54)			Lower in preterm SGA		sSDMA: Higher in preterm-SGA	Office BP: no difference
Holzer et al., 2019 [42]	30 ± 2.3 weeks	5–10 years	VLBW (=44) Term (=30)		No difference	No difference	No difference	sNGAL:no difference, sCystatin:no difference	Office BP: no difference
South et al., 2019 [43]	27.8 ± 2.6 years	14 years	Preterm-VLBW (=96) Term (=43)			Lower in preterm	Higher in preterm		Office BP: SBP, DBP: higher in preterm
Staub et al., 2020 [44]	<32 weeks	10–15 years	Preterm (=51) Term (82)		Higher in preterm	No difference		sNGAL, suUromodulin, sCystatinC, uNGAL, sB2 microglobulin: no difference	Office BP: SBP higher in preterm boys
Mah et al., 2020 [45]	28–36 weeks	6–11 years	Preterm (=125) Term (=250)				69,6% of preterm versus 33,2% of term had proteinuria		BP office:17,6% of preterm had high BP versus 2% of term
Kandasamy et al., 2020 [35]	<28 weeks	6, 12, 24 months	Preterm (=53), Term (=31)	Smaller in preterm		No difference			Office BP: no difference
Restrepo et al., 2022 [36]	32 weeks	5 years	Preterm (=89) Term (=33)	Smaller in preterm. No difference after correction for BSA	No difference	No difference			Office BP: no difference

Abbreviations: ELBW: extremely low birth weight, VLBW: very low birth weight, RL: renal length, RV: renal volume, eGFR: estimated glomerular filtration rate, BUN: blood urea nitrogen, s: serum, u: urine, BP: blood pressure, SGA: small for gestational age, AGA: appropriate for gestational age, BSA: body surface area, ABPM: ambulatory blood pressure monitoring, SBP: systolic blood pressure, DBP: diastolic blood pressure.

**Table 2:** Studies examining the association of prematurity with arterial stiffness during childhood and adulthood.

Author, Year	Age at Assessment	Study Groups (=Sample Size)	Method of Assessment Arterial Stiffness	Device	Results (PWV)
Oren et al., 2003 [57]	28.2 ± 0.9 years	Preterm (=26) Controls (=395)	Carotid radial PWV	SphygmoCor	PWV showed inverse association with GA
Cheung et al., 2004 [58]	8.2 ± 1.7 years	Preterm (SGA = 15, AGA = 36) Controls (=35)	Carotid radial PWV	Photoplethysmography	Higher PWV in preterm SGA
Bonamy et al., 2005 [59]	16.5 ± 0.3 years	Preterm (=34) Term (=32)	Forearm PWV	Photoplethysmography	No difference
Lazdam et al., 2010 [60]	20–30 years	Preterm (=71) (19 hypertensive, 52 normotensive pregnancy), Controls (=38)	cf-PWV	SphygmoCor	Preterm born to normotensive pregnancy had higher PWV than term, Preterm born to hypertensive pregnancy had same PWV with term
McEniery et al., 2011 [61]	11 years	Preterm (=68) Term (=90)	cf-PWV	SphygmoCor	No difference
Rossi et al., 2011 [62]	13–14 years	Preterm (SGA = 25, AGA = 41) Controls (=41)	Carotid radial PWV	Complior	Higher PWV in preterm SGA
Tauzin et al., 2014 [63]	21 years	Preterm (=16) Term (=15)	Carotid radial PWV	Complior	Higher PWV in preterm
Boardman et al., 2016 [64]	23–28 years	Preterm (=102) Controls (=102)	cf-PWV, Bf-PWV	SphygmoCor Vicorder	Higher PWV in preterm
Stock et al., 2018 [65]	15–16 years	Preterm (=83) Controls (=847)	cf-PWV	Complior	No difference in PWV
Kowalski et al., 2018 [66]	18 years	Preterm (=76) Controls (=42)	Carotid radial PWV	SphygmoCor	No difference in PWV
Flahault et al., 2020 [67]	23 years	Preterm (=86) Controls (=85)	cf-PWV	SphygmoCor XCEL	No difference in PWV
Kerkhof et al., 2021 [68]	18–24 years	Preterm (=172) Term (=277)	cf-PWV	SphygmoCor	No difference in PWV
Chainoglou et al., 2022 [69]	10.48 ± 3.8 years	Preterm (=52) Term (=26)	cf-PWV	SphygmoCor XCEL	Higher PWV in preterm

Abbreviations: PWV: pulse wave velocity, GA: gestational age, SGA: small for gestational age, AGA: appropriate for gestational age, cf: carotid-femoral, Bf: branchial-femoral.

## Data Availability

Not applicable.
